# Advances on Aptamers against Protozoan Parasites

**DOI:** 10.3390/genes9120584

**Published:** 2018-11-28

**Authors:** Juan David Ospina-Villa, César López-Camarillo, Carlos A. Castañón-Sánchez, Jacqueline Soto-Sánchez, Esther Ramírez-Moreno, Laurence A. Marchat

**Affiliations:** 1 Grupo BCEI, Universidad de Antioquia, Medellín 050010, Colombia; juando12358@gmail.com; 2 Sección de Posgrado en Ciencias Genómicas, Universidad Autónoma de la Ciudad de México, Ciudad de México 06720, Mexico; cesar.lopez@uacm.edu.mx; 3 Hospital Regional de Alta Especialidad, Oaxaca 71294, Mexico; ccastanon@hraeoaxaca.gob.mx; 4 Sección de Estudios de Posgrado e Investigación, ENMH, Instituto Politécnico Nacional, Ciudad de México 07738, Mexico; jakysotos@yahoo.com.mx (J.S.-S.); estherramirezmoreno@yahoo.com (E.R.-M.)

**Keywords:** aptamers, SELEX, parasites, diagnosis, treatment

## Abstract

Aptamers are single-stranded DNA or RNA sequences with a unique three-dimensional structure that allows them to recognize a particular target with high affinity. Although their specific recognition activity could make them similar to monoclonal antibodies, their ability to bind to a large range of non-immunogenic targets greatly expands their potential as tools for diagnosis, therapeutic agents, detection of food risks, biosensors, detection of toxins, drug carriers, and nanoparticle markers, among others. One aptamer named Pegaptanib is currently used for treating macular degeneration associated with age, and many other aptamers are in different clinical stages of development of evaluation for various human diseases. In the area of parasitology, research on aptamers has been growing rapidly in the past few years. Here we describe the development of aptamers raised against the main protozoan parasites that affect hundreds of millions of people in underdeveloped and developing countries, remaining a major health concern worldwide, i.e. *Trypanosoma* spp., *Plasmodium* spp., *Leishmania* spp., *Entamoeba histolytica,* and *Cryptosporidium parvuum*. The latest progress made in this area confirmed that DNA and RNA aptamers represent attractive alternative molecules in the search for new tools to detect and treat these parasitic infections that affect human health worldwide.

## 1. Introduction

Infectious diseases due to protozoan parasites affect hundreds of million people in underdeveloped and developing countries, remaining a major health concern worldwide. These parasitic infections are usually transmitted through insect vectors or contaminated food and water, and their high prevalence rates are mainly related to poor sanitary conditions or immunodeficiency status. Nowadays, the main methods of diagnostics include the growth and microscopy observation of pathogens from clinical samples, immunodetection of parasite proteins using antibodies, or identification of the pathogen genome by PCR. However, these techniques have several limitations since they require time, low temperature storage conditions, and specialized instruments that are sometimes difficult to get in regions where most infected populations live. Drugs currently used for treating parasite infections are not always effective and some of them have serious side effects; moreover the problem of drug resistance is still a potential threat. Consequently, a large body of biomedical research efforts focused on the identification of new targets and new molecules for parasite control. In this context, aptamers have emerged as attractive tools for the development of alternative diagnostics and treatment methods.

Aptamers are single-stranded RNA (ssRNA) or single-stranded DNA (ssDNA) oligonucleotides whose unique three-dimensional structure enables them to interact with a specific target. Their story begins in 1990 when three independent research groups discovered that nucleic acids, apart from their role in replication, transcription and translation, have the ability to fold in a unique way with the possibility of interacting with virtually any type of target. Robertson and Joyce published a paper where they describe a technique that can be used to evolve a novel function from a natural ribozyme: they isolated a mutant form of the celebrated RNA-splicing *Tetrahymena* ribozyme that is capable of cleaving DNA efficiently [1]. Ellington and Szostak were interested in estimating the number of classes of different kinds of structures capable of interacting with or carrying out a catalytic function, citing as an example the structure of hundreds of proteases that are grouped into only five groups, in order to understand how the origin and evolution of life takes place. For that, they use RNA molecules that turned out to have great potential. To differentiate them from traditional nucleic acids, they were given the name “aptamer” which was coined by Andy Ellington. It stems from the Latin terms “aptus” meaning to fit, and the Greek term “mere” meaning molecule [2]. On the other hand, Tuerk and Gold described for the first time a strategy where a pool of RNA fragments is synthesized to identify proteins that bind to them as part of their evolutionary function. This protocol, which was named as SELEX in reference to the acronym of Systematic Evolution of Ligands by EXponential enrichment later became the standard molecular technique to obtain aptamers. Tuerk and Gold described the ability of RNA molecules to recognize substrates and adapt to fulfill varied enzymatic functions in primordial life forms; they proposed that these extinct RNA molecules could recognize many substrates and act or replace the function of other enzymes, and then they specialized and became more specific, foretelling that “SELEX may be just the beginning of evolution in a test tube” [3].

Like monoclonal antibodies, aptamers specifically recognize and bind to their target. However, there are some interesting differences that make aptamers more suitable tools at detecting and inhibiting target molecules in diagnostics, therapeutics, and drug development (Table 1).

Synthesis of aptamers is only chemical which makes it easy to scale and less susceptible to contamination, while monoclonal antibodies require animal models or cell cultures. The targets identified by antibodies are limited to molecules that are highly immunogenic (usually proteins), while aptamers can recognize different types of molecules regardless of whether they are immunogenic or not, as for example proteins, toxins, metal ions, as well as whole living cells and even tissues [4,5,6,7]. In addition, using RNA molecules to recognize DNA binding proteins and vice versa can contribute to increase aptamers affinity.

One of the great disadvantages of aptamers is their susceptibility to degradation by nucleases and their rapid elimination from the body. However, this problem can easily be overcome thanks to the current facility to introduce chemical modifications at their 3′ and 5′ ends, or add molecules such as polyethylene glycol (PEG) and biotin to prevent degradation. On the other hand, a disadvantage of monoclonal antibodies is their susceptibility to changes in temperature or pH, and once denatured they cannot be repaired. Once selected aptamers are sequenced, they can be chemically synthesized any time it is necessary; they can be lyophilized, they are stable at room temperature, and they can be easily renatured if denatured. The small size of aptamers allows them to reach smaller compartments and cells where antibodies cannot get. In addition, they achieved a better tissue penetration from the injection site. Thinking about the efficiency of scientific production, aptamers consume less production time (one to three months) than antibodies (four to six months) [8,9,10].

The advantages of aptamers over monoclonal antibodies and their huge potential for therapeutic and diagnostic applications rapidly boosted investigations on aptamers in the biomedical field. In 2004, the US FDA (United States Food and Drug Administration) approved the first aptamer-based drug for age-related macular degeneration (AMD) in human. Pegaptanib sodium/Macugen (Pfizer Manufacturing Belgium NV, Puurs, Belgium) is a modified RNA aptamer that targets the vascular endothelial growth factor (VEGF)-165 whose expression leads to pathological ocular neovascularization and vascular permeability [11]. Our search at the https://clinicaltrials.gov/ct2/home  Clinical trials web site [12] revealed that more than 30 undergoing clinical trials involving aptamers are currently being carried out: one is in phase IV (Pegaptanib-approved), seven are in phase III, 13 in phase II, 14 in phase I, and 1 in early phase I. These aptamers were designed for the treatment of a variety of human diseases, including neovascular AMD, diabetes mellitus type II, Leucemia Mieloide Aguda (LMA), or hemophilia, among others.

Currently, aptamers are considered as molecules that have a great potential and multiple applications similar to monoclonal antibodies. Larry Gold mentioned in 2015 in a special issue that summarized the 25 years of the discovery of the aptamers “…the future for applications of aptamers will be limited only by our imagination, as is always the case. Already aptamers have been used for proteomics, cell sorting, pathology, affinity purification, and pharmaceuticals, and those are just the things that build on prior work with antibodies” [13]. In the area of parasitology, investigation on aptamers is still in the early stages, but promising results have been obtained in several protozoan parasites that affect human health. Malaria caused by *Plasmodium* species (spp.) is the most prevalent infection worldwide among vector-borne parasitic diseases, with more than 210 million cases [14]. Leishmaniasis affects about 12 million individuals in 88 countries of four continents; symptoms depend on the *Leishmania* species and range from skin ulcers (*L. major*, *L. tropica*, *L. braziliensis*, *L. mexicana*) to mucocutaneous (*L. braziliensis*) and visceral (*L. donovani*, *L. chagasi*, *L. infantum*) lesions. About 0.5 and 10 million people are infected with *Trypanosoma brucei* and *T. cruzi* that causes African (or sleeping sickness) and American (or Chagas disease) trypanosomiasis, which are transmitted by the bite of an infected tsetse fly and triatomine bug, respectively [15]. The intestinal protozoan *Entamoeba histolytica* that is responsible for human amoebiasis is considered as the third cause of death from parasites, after *Plasmodium* and the helminthic worm *Schistosoma*; it has been estimated that *E. histolytica* infects 10% of the world population, causing about 100,000 deaths each year [16]. The opportunistic *Cryptosporidium parvuum* is considered as an emerging health public problem, being a very common cause of gastroenteritis; it also provokes severe diarrhea in immunocompromised people, mainly in young children and human immunodeficiency virus (HIV) –infected individuals [17]. In this review we describe some technical aspects of DNA/RNA aptamers design and modifications, including data about the different approaches used for the selection of parasite-specific aptamers. We also present a panorama of the potential applications of these aptamers, particularly as valuable tools for the development of new diagnosis methods, new drugs, as well as new tools for drug delivery and fundamental research. Altogether, these data confirmed the great potential and multiple applications of aptamers to detect and control some of the most challenging protozoan parasites for human health.

## 2. Generation and Modification of DNA/RNA Aptamers

### 2.1. SELEX for DNA/RNA Parasite-Specific Aptamer Selection

**Figure 1:**
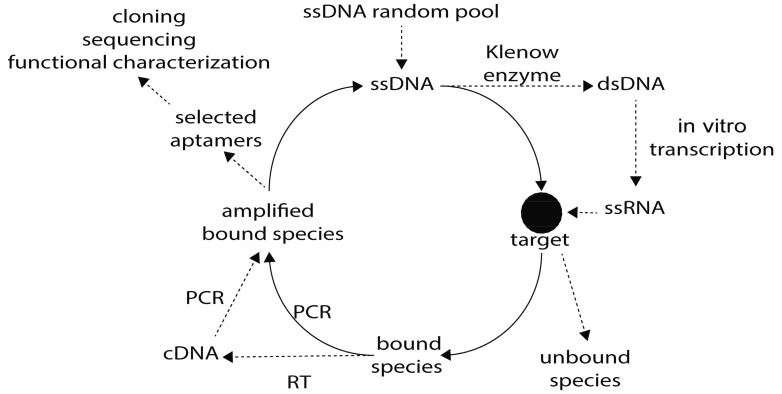
Graphical representation of the SELEX (Systematic Evolution of Ligands by EXponential enrichment) strategy. The initial library of single-stranded DNA (ssDNA) may be used immediately for interaction with the target, while the ssRNA library is obtained after synthesis of complementary strand by Klenow enzyme followed by in vitro transcription. After interaction with the target using different strategies, unbound molecules are discarded while bound oligonucleotides are PCR amplified for a new round of SELEX. After a dozen rounds to improve specificity, selected aptamers are cloned into a plasmid to be sequenced. PCR: polymerase chain reaction, cDNA: complementary DNA, RT: reverse transcription, dsDNA: double stranded DNA

The SELEX strategy in its simplest form could be summarized in three fundamental steps: (1) incubation of a random nucleic acid library (a set of 10^12^ to 10^15^ combinatorial oligonucleotides that consist in a random sequence region, usually 30–40 mers, flanked by two conserved primer sites) with the target (from ions to whole cells or tissues); (2) selection of aptamers that recognize the target and elimination of those that do not; and (3) amplification of selected aptamers using conserved primer sequences for further rounds of selection. For the generation of DNA aptamers, the DNA library can be tested directly against the selected target, while using an RNA aptamer requires an additional step where double-stranded (ds) DNA must be obtained and subsequently transcribed to create the ssRNA library. So to obtain RNA aptamers, more time and money is required [18] (Figure 1). An additional but fundamental step is the one known as “negative selection” in which aptamers that do not recognize an alternative target are kept, while those that do are discarded. In general, this optional step significantly improves the specificity of the selected aptamers and allows the design of a personalized strategy [8]. In the field of parasitology, the original SELEX protocol has been successfully used to develop aptamers that bind to specific proteins of *Leishmania* spp. [19,20,21,22], *Plasmodium* spp. [23,24,25,26] and *E. histolytica* [27].

The SELEX strategy has evolved over the years becoming more and more efficient, and being directed against increasingly complex targets. One of the most frequently used variants for parasite-specific aptamer selection is the cell-SELEX method, in which targets are proteins in the external cell surface of whole living cells. The main advantage of this method is that targets do not have to be known, purified and immobilized; moreover, targets with post-translational modifications can be detected. Depending on whether the cells are adherent or not, the removal of unattached aptamers is done by means of washing (adherents) or centrifugation (suspension). Aptamers obtained from cell-SELEX strategy are thought to be useful for cell-specific diagnosis, cell-targeted drug delivery, and cell-specific therapy [28]. They can also be used for disease cell recognition [29]. Importantly, a cell-SELEX-derived aptamer has more possibility to be used directly for in vivo and clinical applications [30]. Therefore, the cell- SELEX strategy has been used to generate aptamers with affinity to surface components of *Trypanosoma* spp. [31,32,33,34] and *C. parvuum* [35] with the aim of developing new diagnosis tools or blocking host–parasite interaction.

Other types of SELEX have been developed, all seeking to adapt and improve the SELEX capacity for parasite-specific aptamers selection. Notably, Liang and Connell adapted the SELEX protocol using a streptavidin-coated microtiter plate filled with RNA fragments to study RNA editing in *Leishmania* spp. [36]. Moreno et al. designed a novel SELEX methodology using colloidal gold to select high affinity single stranded DNA aptamers against a *Leishmania* protein [37], and Birch et al. used an inertial microfluidic SELEX approach to isolate aptamers with affinity to several surface-displayed epitopes on erythrocytes infected with *Plasmodium* spp. [38].

### 2.2. Modifications of Parasite-Specific Aptamers to Increase their Efficacy

Despite of the multiple advantages of aptamers over monoclonal antibodies and their large range number of potential uses, one of the most difficult part for the development of this technology is to achieve that aptamers that have in vitro activity can also be useful in vivo. The main issues are their rapid renal excretion and degradation by nucleases. Fortunately, aptamers can be chemically modified to prevent these problems. Phosphodiester backbone modifications, sugar ring modification and 3′ end capping have been shown to avoid nuclease degradation. On the other hand, successful strategies have been implemented for resisting renal clearance, such as 5′ end polyethylene glycol (PEG)ylation, and 3′ biotin-streptavidin conjugation. Such changes also seek to increase aptamer solubility, melting temperature and stability. These modifications can be included directly in the libraries, although some difficulties have been reported with DNA polymerase when processing modified nucleotides. They can also be incorporated at the end of the SELEX strategy, once the aptamers have been obtained, with the risk of altering the affinity and specificity of aptamers [8,39,40].

Regarding aptamers with affinity to protozoan parasites components, some of these modifications have been demonstrated to improve their efficacy (Table 2). While the addition of high molecular mass PEG molecules impaired the functionality of aptamer 2–16 that recognizes a 42 kDa protein located in the flagellar pocket of the infective blood life cycle stage of the *T. brucei*, the conjugation with smaller molecules such as 2′-NH_2_, 2′-deoxy-2′-F, 2′-deoxy-2′-NH_2_-uridine, 2′-deoxy-2′-NH_2_-cytidine, or small PEG polymers changed it into a serum-stable and functional RNA aptamer, providing interesting data for its potential therapeutic application in vivo [41,42]. RNA aptamers that bind surface components of *T. brucei* and recognize *T. cruzi* proteins in the plasma of infected mice have been conjugated to biotin with the aim of labelling and detecting parasites for diagnosis purposes [32,43].

## 3. A Few Words about Peptide Aptamers

As described above, originally, aptamers are short ssDNA or RNA oligonucleotides that are capable of binding various molecules with high affinity and specificity. However, in the period after their discovery, the use of peptides as potential aptamers that are also known as “paptamers” was implemented. Peptide aptamers are artificial recognition molecules that consist of a variable peptide sequence inserted into a constant scaffold protein. Peptide aptamers are different from other classes of proteins because they have small size, simple design and independent folding.

A great difference between aptamers based on nucleic acids and peptides is the method of obtaining. The SELEX strategy is the classic method for obtaining DNA or RNA aptamers, while the paptamers are obtained by means of yeast two-hybrid systems. The excellent recognition specificity and high binding affinity typical of peptide aptamers have suggested that they could be used in many protein detection methods. Today, they are used in microarrays for proteins function characterization, protein–protein interaction assays, protein structure evaluation and to inhibit protein function. Here, we will not deepen on peptide aptamers but excellent works on these molecules can be consulted [44,45,46,47]. To our knowledge, the only report about peptide aptamers in protozoan parasites is related to the development of a 44 amino acid long peptide whose binding to RAD51 paralogues led to DNA damaging agent sensitivity and inhibited *T. brucei* proliferation [48].

## 4. Development of DNA/RNA Aptamers in Parasitology

**Figure 2:**
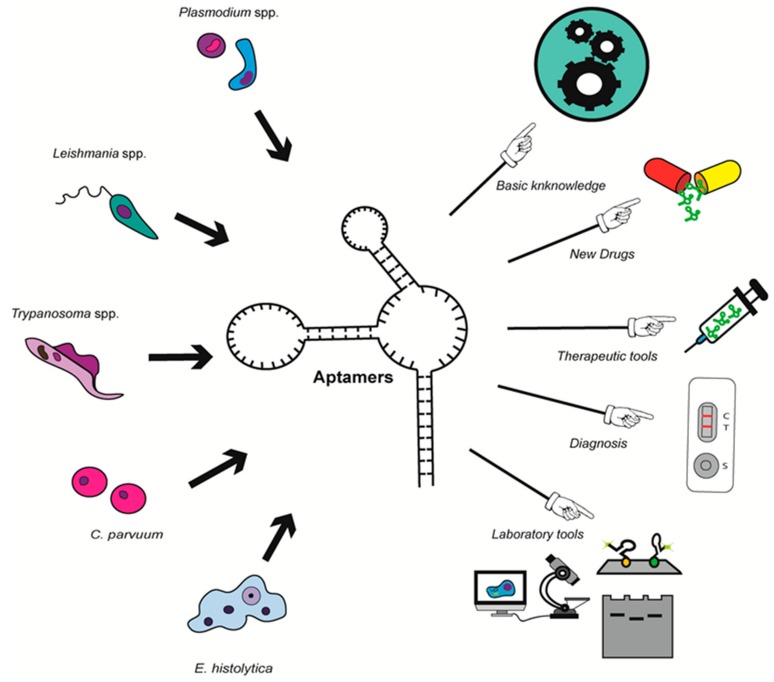
Potential applications for DNA/RNA aptamers against proteins of *Plasmodium* spp., *Leishmania* spp., *Trypanosoma* spp., *C. parvuum* and *E. histolytica*.

The number of works on parasite-specific aptamers has greatly increased in the last decade, together with their potential use for different applications. In most studies, aptamers are efficient molecules for parasite detection, including in aptamer-based biosensors; in others, aptamers are designed as new drugs able to block parasite survival or infection process; additionally, they can also represent new tools for drug delivery, as well as molecular mechanisms study and protein purification in fundamental research. To date, the state of the art only includes aptamers that recognize proteins of *Trypanosoma* spp., *Leishmania* spp., *Plasmodium* spp., *E. histolytica* and *C. parvuum* that are some of the most challenging protozoan parasites for human health (Figure 2 and Table 3).

### 4.1. Aptamers against Trypanosoma spp.

To our knowledge, the first report on aptamers against protozoan parasites that affect human health goes back to 1999. Surface components of Trypanosomes have always represented attractive markers for parasite detection in blood of infected individuals; their role in parasite adhesion and entry into the cell also make them interesting targets to block infection. In this context, the group of Göringer used the cell-SELEX strategy to select RNA aptamers with high affinity for a 42 kDa protein located in the flagellar pocket of the infective blood life cycle stage of the *T. brucei* [31]. Additional characterization of one of these aptamers, the so-called aptamer 2–16, revealed that after binding to its target, the aptamer is partially processed to a 50 nucleotide long molecule, internalized by receptor-mediated endocytosis and transported to the lysosome. Taking advantage of these processes, the authors showed that aptamer 2–16 can be used as a ‘piggy-back‘ delivery trypanocidal molecule to target the lysosomal compartment of trypanosome, revealing that aptamer 2–16 may represent an attractive therapeutic tool to facilitate drug delivery to lysosome [49]. Other relevant proteins of the parasite protein surface are the variant surface glycoproteins (VSG) that allow trypanosomes to escape the immune response of the infected host by antigenic variation. Using the structurally conserved C-end of VSG molecules, Lorger et al. obtained RNA aptamers that bind the surface of live African trypanosomes. When attached to an antigenic molecule, these aptamers can function as therapeutic tools to direct antibodies to the surface of trypanosome cells, promoting parasite elimination [32].

Notably, most aptamers related to *Trypanosoma* spp. have potential for parasite diagnosis. Thus, aptamers described above can be biotinylated to provide a new strategy to label *T. brucei* membrane [32]. Therefore, they were used in a potentiometric carbon-nanotube system to develop an aptasensor that detects parasites in blood samples [50].

The PCR detection of trypomastigotes in blood is sometimes difficult due to their low concentration. So, in order to propose an alternative for diagnosis, Nagarkatti et al. used the cell SELEX protocol to obtain aptamers that bind to *T. cruzi* trypomastigotes with high affinities (Dissociation constant (Kd) of 30 nM). Notably, aptamer Apt68 that showed the highest binding to live parasites also binds to trypomastigotes from other parasite strains, indicating that its target is a conserved component of the mammalian parasite form. Interestingly, Apt68 did not show a cross reactivity for insect stage epimastigotes of *T. cruzi* neither for other trypanosomatid parasites, namely *Leishmania donovani* and *T. brucei*, which is a huge advantage for a diagnostic tool. Therefore, the authors developed a system based on magnetic beads coated with aptamer that could be used to capture parasites at low concentration in blood to facilitate their PCR-based detection [34].

In another work, the same group reported aptamers can be used to detect parasite proteins called *Trypanosoma* Excreted Secreted Antigens (TESA) that are released in the plasma of *T. cruzi* infected mice. Notably, the biotinylated aptamer L44 (Apt-L44) also recognized TESA in *T. cruzi* trypomastigote extract, but not in mouse or *Leishmania* proteins in an Enzyme Linked Aptamer Assay (ELAA). Because Apt-L44 recognizes a conserved target in different strains of *T. cruzi* and can detect them in the plasma of infected mice, both in the acute and chronic phases, the authors proposed that it could represent a new tool for detecting biomarkers of Chagas disease [43].

*Trypanosoma*-specific aptamers can also represent new drugs to control this parasitic disease. Because the interaction of *T. cruzi* trypomastigotes with extracellular matrix (ECM) components is vital for the establishment of infection in the host cell, the group of Alves performed a cell-SELEX protocol to develop RNA aptamers that bind with high affinity and specificity to receptors of host ECM molecules on *T. cruzi*, using laminin, fibronectin, heparan sulfate, or thrombospondin as a displacement agent. In agreement with the importance of these molecules in mediating the infection process, selected aptamers were able to inhibit parasite invasion of LLC-MK2 monkey kidney cells by 50%–70% in vitro [33].

### 4.2. Aptamers against Leishmania spp.

In *Leishmania*, another hemoflagelate of the Trypanosomatidae family, a few groups have designed specific aptamers to elucidate some molecular and cellular events in parasites. In 2002, Bhattacharyya et al. used the SELEX protocol to identify the sequences in cytoplasmic transfer RNA (tRNA) that are recognized by import receptors of mitochondria. Incubation of purified *L. tropica* mitochondria with a combinatorial RNA library led to the selection of a variety of import aptamers containing sequence motifs present in the anticodon arm, the D arm, the V-T region, and acceptor stem of known tRNAs. Functional assays of a subset of aptamers showed that they have nanomolar affinities for mitochondria and revealed the existence of two groups of RNA molecules with distinct intrinsic efficiencies of transfer across the outer and inner mitochondrial membranes. Their interaction with each other at the inner membrane also suggested the existence of an allosteric regulation at the inner membrane receptor complex in order to maintain a properly balanced tRNA pool for mitochondrial translation [19].

Another attractive molecular mechanism in *Leishmania* is RNA editing which consists in abundant uridine insertions and deletions in mitochondrial-encoded transcripts. Liang and Connell in 2009 adapted the SELEX protocol to detect editing activity in a mitochondrial fraction from *L. tarentolae*. They filled in a streptavidin-coated microtiter plate with a pool of 94-nt (nucleotides) RNA fragments containing 21 randomized positions flanked by key elements of the streptavidin binding and editing substrate, and incubated it with mitochondrial extracts. Edited RNA aptamers were activated by editing-induced conformational changes detectable through an electrochemiluminescent signal. Biochemical measures confirmed that this aptamer-based assay is highly sensitive detecting the edited products in the femtomole range, which suggests its possible use for the evaluation of other RNA processing reactions [36].

Besides the development of aptamers that can be used as tools to elucidate parasite *Leishmania* molecular mechanisms, other groups have generated aptamers with diagnosis purposes. The group of Gonzalez focused on the kinetoplastid membrane protein-11 (KMP-11), a cytoskeleton-associated protein that is thought to participate in mobility of the flagellar structure. By using a colloidal gold-based SELEX methodology, Moreno et al. (2003) selected ssDNA aptamers against *L. infantum* KMP-11. Their specificity was further confirmed by Dot blot, ELISA (Enzyme-Linked ImmunoSorbent Assay) and Western blot experiments. These initial results opened the way for the identification of aptamers against parasite protein that could be used to detect *Leishmania* infection [37].

Indeed, the same group has used the SELEX protocol to select aptamers that recognize several specific nuclear proteins in *Leismania*. Even though histones are extremely conserved proteins, there are high sequence divergences in the N- and C-terminal domains in kinetoplastid proteins, turning them into interesting targets. For this reason, Ramos et al. obtained a ssDNA aptamer population which binds to *L. infantum* H2A with high affinity and does not recognize other *Leishmania* proteins neither other histones, according to ELONA (Enzyme-Linked OligoNucleotide Assay), slot blot and Western blot assays [20]. Two of these aptamers named as AptLiH2A#1 and AptLiH2A#2 were shown to specifically recognize the LiH2A protein into complex mixtures with Kd values that correspond to the best range of most of the aptamers previously described. Although they show distinct primary and secondary structures among them, both aptamers targeted the same peptides in the LiH2A protein. Interestingly, both aptamers were able to identify recombinant and native parasite H2A proteins; they were also useful to determine the number of parasites in an ELONA platform, which demonstrated their potential for a diagnosis marker. They can also be useful as a laboratory tool with purification purpose [51]. Other DNA aptamers with high affinity and specificity for *L. infantum* histone H3 may also have a potential application to detect leishmaniasis [21].

Considering the importance of RNA posttranscriptional maturation events for gene expression regulation in *Leishmania*, the group of Gonzalez recently developed aptamers targeting the poly (A)-binding protein (PABP) that binds the 3′ end of mRNA poly(A) tail and participates in translational initiation and termination, and in messenger RNA (mRNA) turnover in eukaryotic cells, making it a key protein for the survival of the parasite. The SELLiPABP aptamers population obtained by SELEX bound to LiPABP with high affinity (Kd = 3.87 ± 0.67 nM). Particularly, three isolated aptamers (ApPABP#3, ApPABP#7 and ApPABP#11) were able to recognize specifically the recombinant protein with affinity in the low nanomolar range, making them useful for the development of diagnostics systems for leishmaniasis. They were also able to detect LiPABP from only 2500 parasites, which is a promising result for the development of a diagnosis method. Interestingly, functional assays revealed that one of these aptamers, ApPABP#11, disrupts the binding of LiPABP to poly(A) tail, suggesting that it may affect parasite gene expression in vivo. This ability may be used to regulate the function of LiPABP. In addition, the three aptamers were efficient tools to purify LiPABP from complex mixtures [22].

### 4.3. Aptamers against Plasmodium spp.

Surprisingly, the first report on *Plasmodium*-specific aptamers dates from 2009, i.e., 10 years after the first article on parasite-specific aptamers, when Barforld et al. reported the utilization of the SELEX protocol to obtain RNA aptamers against the semiconserved DBL1α region of the *P. falciparum* erythrocyte membrane protein 1 (PfEMP1) involved in parasite adhesion to blood vessels and erythrocytes, showing their potential for altering host–parasite interaction and therefore their pathogenicity [23]. Since then, the number of papers about aptamers against *Plasmodium* proteins has largely increased, seeking the development of new diagnosis methods as the main aim. Most of them target the lactate dehydrogenase (LDH), which is a known biomarker of parasitaemia. In 2013, taking advantage of sequence and structural divergence between the parasite and mammalian LDH, the group of Tanner identified the 2008s ssDNA aptamer which has a high affinity for the *P. falciparum* enzyme (PfLDH), with a dissociation constant of 42 nM and a 2:1 protein:aptamer stoichiometry. Analysis of the complex crystal structure revealed that aptamer specificity is related to its interaction with a LDH loop that is absent in the human protein. Interestingly, the enzymatic activity was maintained in the complex which allowed the development of an aptamer-tethered enzyme capture (APTEC) colorimetric assay for the rapid diagnosis of malaria in blood samples through the detection of PfLDH [24,52]. Previously, using the *P. vivax* LDH as target, the group of Ban obtained the pL1 ssDNA aptamer and designed an apL1 aptamer-based electrochemical sensor capable of discriminating malaria positive samples (*P. vivax* and *P. falciparum*) from non-infected samples [25]. Interestingly, pL1 has a different sequence and structure than the 2008s aptamer, and its PvLDH recognition that involves predominant shape complementarity with many bridging water molecules, is totally different from that of 2008s [53]. Later, the group of Goswani described the P38 ssDNA aptamer that recognizes the PfLDH with a dissociation constant of 0.35 µM [26].

Several nanotechnologies tools were assessed to strengthen the application of 2008s, pL1 and P38 aptamers for malaria diagnosis, such as gold nanoparticles [24,25,54,55,56], DNA origami [57,58], DNA tweezer [59], magnetic microparticles [60,61], MoS_2_ nanosheet [62,63] and silver nanoclusters [64], demonstrating the relevance of these aptamer-based sensors (aptasensors) for simple and sensitive detection of parasites in clinical blood samples.

Among the five *Plasmodium* species that are known to infect humans, *P. falciparum* and *P. vivax* display the highest prevalence, but *P. falciparum* is the most virulent species. Without a rapid accurate treatment, *P. falciparum* infection may worsen and be fatal. With the aim of developing a highly specific test for malaria, two recent works described aptamers capable of precisely distinguishing *P. falciparum* from other species. Using an APTEC assay, Cheug et al. showed that the previously described aptamer 2008s was specific for *P. falciparum* and could discriminate against *P. vivax* in malaria patient blood samples [65]. Frith et al. took advantage of the LISDAELEAIFDC epitope that is unique to the *P. falciparum* LDH, to develop the LDHp 11 aptamer that detects only PfLDH and not PvLDH [66].

In addition to their potential for malaria diagnosis, aptamers represent potential therapeutic molecules. In 2009, Niles et al. used heme-binding DNA aptamers that inhibit hemozoin formation in vitro, in a similar way as the anti-malarial compound chloroquine acts. Consistently, the uptake of red blood cells loaded with heme-binding aptamers resulted in parasite toxicity [67]. By an inertial microfluidic SELEX approach, Birch et al. isolated several surface-displayed epitopes on parasite-infected RBCs (Red Blood Cells), including an aptamer against the protein responsible for placental sequestration, var2CSA [38].

### 4.4. Aptamers against Cryptosporidium parvuum

The use of aptamers for the study of *C. parvuum* has not been widely addressed and the only published work aimed to design a specific and sensitive method for detecting the presence of *Cryptosporidium* in foods, which represent one of the main ways of infection. Using a cell-SELEX protocol, Iqbal et al. (2015) selected 14 ssDNA aptamers able to bind to the oocyst wall of *C. parvuum*. To confirm their affinity for oocysts, they developed an electrochemical detection protocol in which each aptamer was hybridized to a thiol-modified complementary primer and immobilized onto a gold nanoparticles-modified screen-printed carbon electrode (GNPs-SPCE). After interaction with *C. parvuum* oocysts, one aptamer named R4–6 was shown to exhibit the highest binding affinity to the oocysts as demonstrating by the major increase in the redox current measured by square wave voltammetry. This aptasensor was able to detect oocysts in the range of 150 to 800 oocysts, with a detection limit of approximately 100 oocysts. Interestingly, the aptasensor was able to distinguish between *C. parvuum* and other parasites, e.g., *Giardia duodenalis* in pineapple and mango concentrates. The authors concluded that the high sensitivity and specificity of the aptasensor make it a promising tool for the detection and identification of *C. parvuum* oocysts in foods [35].

### 4.5. Aptamers against Entamoeba histolytica

The only work about aptamers against *Entamoeba histolytica* proteins came from our group. Polyadenylation of messenger RNAs is a fundamental step for the regulation of gene expression in eukaryotic cells. For several years, we have been investigating RNA sequences in 3′ UTR and proteins involved in this process in *E. histolytica* trophozoites. Particularly, we performed the molecular and functional characterization of the 25 kDa subunit of the Cleavage Factor Im (EhCFIm25) whose homologue participates in poly(A) site selection, polyadenylation factors recruitment, RNA cleavage and poly(A) tail synthesis in eukaryotic cells [68,69,70]. Notably, we showed that EhCFIm25 is essential for parasite survival and virulence, since its silencing by specific dsRNA reduced parasite mobility, and erythrophagocytosis capacity, and induced cell death [71]. Then, we used the SELEX strategy for two purposes: first, to know the specific RNA sequence bound by EhCFIm25 as it has been done previously with the human protein [72]; and second, to obtain specific RNA aptamers that recognize the EhCFIm25 protein. After seven rounds of SELEX, we selected RNA aptamers that share the GUUG sequence. RNA-binding assays further confirmed that EhCFIm25 binds to this motif that is different from the UGUA motif recognized by the human CFIm25 protein. Additionally, introduction of two isolated aptamers (C4 and C5) in trophozoites dramatically inhibited *E. histolytica’s* growth. We hypothesized that C4 and C5 aptamers were able to compete with the GUUG motif contained in endogenous RNA for EhCFIm25 binding; this interaction prevented EhCFIm25 from carrying out its function, producing a non-viable phenotype in vitro [27]. Additional experiments currently in progress are focused on the use of these aptamers in different applications such as an alternative for protein detection and purification by antibodies, or a new tool for parasite diagnosis. Aptamers C4 and C5 may also represent powerful biotechnology instruments to control parasites by blocking polyadenylation and therefore gene expression.

## 5. Conclusions

The numerous advantages of DNA/RNA aptamers regarding design, synthesis, modifications, specificity, stability, as well as time and cost production, make them attractive tools for the developments of new diagnosis and therapeutic methods. Since the expiration of the protection period for the SELEX patent, the number of works on aptamers has exponentially increased which led to the development and clinical evaluation of news therapeutic molecules for a large range of human diseases. The area of parasitology is not the exception and several groups working on *Trypanosoma* spp., *Plasmodium* spp., *Leishmania* spp., *Entamoeba histolytica,* and *Cryptosporium parvuum* have used distinct SELEX approaches to develop aptamers that interferes with host–parasite interaction to block invasion, target intracellular proteins to inhibit parasite survival, or recognize specific markers in blood to detect and quantify parasite infection. Data obtained so far are particularly promising for these parasitic diseases that lacks efficient vaccines and whose diagnosis and treatment are not enough efficient. The latest progresses made in this area clearly point out the utility of aptamers for diagnostic and therapeutic purposes (Figure 2). Consistently, a quick search in https://patents.google.com/ (October 2018) using “aptamer” and “parasite name” as search terms (with “grant” as status and “patent” as type) yields to 15 results for *Plasmodium*, seven for *Leishmania*, 11 for *Trypanosoma*, three for *Cryptosporidium* and two for *Entamoeba*. Even if there is still a long way from basic science towards the routine clinical use of aptamer in the field, there is no doubt that aptamers will have a significant role in a near future.

## Figures and Tables

**Table 1:** Comparison between DNA/RNA aptamers and monoclonal antibodies.

Key Points	Aptamers	Monoclonal Antibodies
Synthesis	Production process is purely chemical	Requires an immune response in an animal model
Target	Almost any type of molecule	Immunological molecule
Modification	Chemical modification to improve resistance to nucleases and bioavailability	Antibodies are typically conjugated with one type of signaling or binding molecule
Stability	Aptamers are fairly stable at ambient temperature and are easily refolded if denatured	Antibodies are susceptible to high temperatures and pH changes; denatured antibodies cannot be repaired
Long-term availability	Once the nucleotide sequence is known, the aptamers can be produced chemically when necessary	Frozen cell stocks must be maintained for monoclonal antibody production
Size	12–30 kDa (~30–80 nucleotides)	~150–170 kDa (IgG)
Production time	~1–3 months	~4–6 months

**Table 2:** Modifications of parasite-specific aptamers for diagnosis and treatment.

Modification	Position	Effect	Parasite/Application	Reference
Sugar modification	2′-OH to 2′-fluorine, 2′-O-methyl	Increases half-life	*T. brucei*/treatment	[40,41, 42]
Conjugation with biotin	-	Blocks activity of 3′ exonucleases	*T. cruzi*/diagnosis	[32,43]
PEGylation	5′conjugation	Increases half-life and solubility, offers protection from reticuloendothelial cells and proteolytic enzymes	*T. brucei*/treatment	[41,42]

PEG: polyethylene glycol.

**Table 3:** Proposed applications for parasite-specific aptamers.

Proposed Application	Nature of Aptamer	Protozoan	Parasite Target	Reference
Diagnosis	RNA	*T. brucei*	VSG proteins	[50]
Diagnosis	RNA	*T. cruzi*	live parasites in blood	[34]
Diagnosis	RNA	*T. cruzi*	TESA	[43]
Diagnosis	DNA	*L. infantum*	KMP-11	[37]
Diagnosis	DNA	*L. infantum*	H2A	[20,51]
Diagnosis	DNA	*L. infantum*	H3	[21]
Diagnosis	DNA	*L. infantum*	PABP	[22]
Diagnosis	DNA	*P. falciparum*	LDH	[24,52,65]
Diagnosis	DNA	*P. vivax*	LDH	[25]
Diagnosis	DNA	*P. falciparum*	LDH	[66]
Diagnosis	DNA	*C. parvuum*	oocyst	[35]
New drug	RNA	*T. cruzi*	Receptor of host ECM molecules	[33]
New drug	DNA	*L. infantum*	KMP-11	[37]
New drug	DNA	*L. infantum*	PABP	[22]
New drug	RNA	*P. falciparum*	EMP1	[23]
New drug	DNA	*P. falciparum*	hemozoin	[67]
New drug	DNA	*P. falciparum*	var2CSA	[38]
New drug	RNA	*E. histolytica*	CFIm25	[27]
Drug delivery	RNA	*T. brucei*	42 kDa protein	[49]
Drug delivery	RNA	*T. brucei*	VSG proteins	[32]
Molecular mechanisms	RNA	*L. tropica*	mitochondria	[19]
Molecular mechanisms	RNA	*L. tarentolae*	mitochondrial extracts	[34]
Protein purification	DNA	*L. infantum*	LiH2A	[20,51]
Protein purification	DNA	*L. infantum*	PABP	[22]

LDH: lactate dehydrogenase; TESA: *Trypanosoma* Excreted Secreted Antigens; PABP: poly(A)-binding protein; VSG: variant surface glycoproteins; KMP-11V: kinetoplastid membrane protein-11; ECM: extracellular matrix; EMP1: erythrocyte membrane protein 1.
